# TT3.1: a journey to protect chloroplasts upon heat stress

**DOI:** 10.1007/s44154-022-00051-4

**Published:** 2022-07-12

**Authors:** Jin-Yu Li, Jian-Xiang Liu

**Affiliations:** State Key Laboratory of Plant Physiology and Biochemistry, College of Life Sciences, Zhejiang University, Hangzhou, 310027 China

**Keywords:** Chloroplast, ER, Heat stress, NTL3, Plasma membrane, TT3.1, TT3.2

## Abstract

Rice (Oryza sativa L.) is a staple crop that feeds over half the world’s population. High temperature stress is a great threaten to sustainable agriculture and leads to yield loss and impaired grain quality in major crops. Rice is sensitive to heat stress at almost all the growth stages and the molecular mechanisms underlying responses to heat stress in rice is emerging. Through quantitative trait locus (QTL) mapping, a recent study conducted by Zhang et al. shows that one genetic locus Thermo-tolerance 3 (TT3) contains two genes that are required for thermotolerance in rice. The TT3.1–TT3.2 genetic module in rice links the plasma membrane to chloroplasts to protect chloroplasts from heat stress damage and increases grain yield under heat stress conditions. This breakthrough provides a promising strategy for future breeding of high temperature resilient crops.

Global warming threatens harvests and has negative impacts on agricultural production worldwide. Chloroplasts are essential for photosynthesis, and their biogenesis and function are tightly regulated by environmental temperatures (Li et al. 2022). Although much has been learned on understanding heat stress response in plants (Ding et al. 2020), the interactions and communications between different subcellular organelles, especially chloroplasts under heat stress conditions are still less understood. A recent study reports a novel quantitative trait locus (QTL) named *Thermo-tolerance 3* (*TT3*) which encodes an E3 ubiquitin ligase TT3.1 and a chloroplast-localized membrane protein TT3.2 to enhance thermotolerance in rice (Zhang et al. 2022). These exciting findings reveal a novel communication mechanism between plasma membrane (PM) and chloroplasts upon heat stress, and provide an efficient strategy for breeding thermotolerant crops.

QTL mapping with chromosome segment substitution lines developed with the African rice variety CG14 as the donor parent and the Asian rice variety Wuyunjing (WYJ) as the recurrent parent leads to the identification of *TT3* (Zhang et al. 2022) and *TT1*, encoding an α2 subunit of the 26S proteasome (Li et al. 2015), both of which are responsible for thermotolerance in rice. The nearly isogenic line (NIL) NIL-*TT3*^*CG14*^ shows more tolerant than the NIL-*TT3*^*WYJ*^ plants under heat stress conditions, and the loss-of-function mutant of *TT3.1* is more sensitive to heat stress while mutation of *TT3.2* confers thermotolerance comparing to the wild-type control WYJ. Overexpression of both *TT3*^*WYJ*^ and *TT3*^*CG14*^ in WYJ background increase thermotolerance. In greenhouse and field trials, either *TT3.1*^*CG14*^ overexpression plants or *tt3.2* mutant plants exhibit a significant increase in seed setting rate, 1000-grain weight and ultimate grain yield per plant, indicating that *TT3* also enhances thermotolerance at reproductive stages. Together, these results indicate that *TT3* locus contain two casual genes that acts in an opposite way for thermotolerance in rice.

TT3.1 is a RING-type E3 ligase with predicted transmembrane domains and it translocates from PM to endosomes in response to heat stress. Coincidentally, TT3.2 shows more endosome localization and less chloroplast localization under heat stress conditions comparing to that under normal temperature conditions. Intriguingly, Zhang et al. (2022) demonstrate that TT3.1 and TT3.2 interacts in yeast two-hybrid and split-luciferase assays. They later found that TT3.1 is a functional E3 ligase that polyubiquitinates TT3.2^WYJ^ and TT3.2^CG14^ *in vitro*. They also found that TT3.1^CG14^ has a stronger activity than TT3.1^WYJ^ *in vivo* in terms of TT3.2 degradation, and this degradation process is mediated by the endosome-to-vacuole pathway.

Since the degradation of TT3.2 upon heat stress is essential for heat tolerance in rice, it prompted the authors to study the molecular function of TT3.2. The results showed that the thylakoid organization of chloroplasts in NIL-*TT3*^*WYJ*^ and *tt3.1* mutant plants is impaired. They also found the abundance of photosynthetic protein complexes and the core subunits of photosystem II (PSII), D1 and D2, are decreased in NIL-*TT3*^*WYJ*^ and *tt3.1* mutant plants under heat stress conditions. These results suggest that the accumulation of chloroplast-localized TT3.2 plays a negative role in the maintenance of thylakoid organization, photosynthetic protein complexes and core subunits of PSII under heat stress conditions. How TT3.2 damages thylakoids and the PSII complex under heat stress conditions is not known. Nevertheless, the current report uncovers a novel mechanism in which TT3.1 travels from PM to endosomes to degrade TT3.2 for protecting chloroplasts upon heat stress, which is essential for thermotolerance in rice.

Heat stress denatures proteins and disrupts protein homeostasis in the cytosol, endoplasmic reticulum (ER), chloroplasts, mitochondria, and even nucleus (Sun et al. 2021). Previously, we reported that a membrane-associated NAC transcription factor NTL3 relocates from PM to nucleus under heat stress conditions to regulated the expression of genes involved in reactive oxidative species (ROS) detoxification and protein folding in ER (Liu et al. 2020). These PM-associated proteins TT3.1 and NTL3 might serve as thermosensors in rice plants (Fig. 1).

**Fig. 1:**
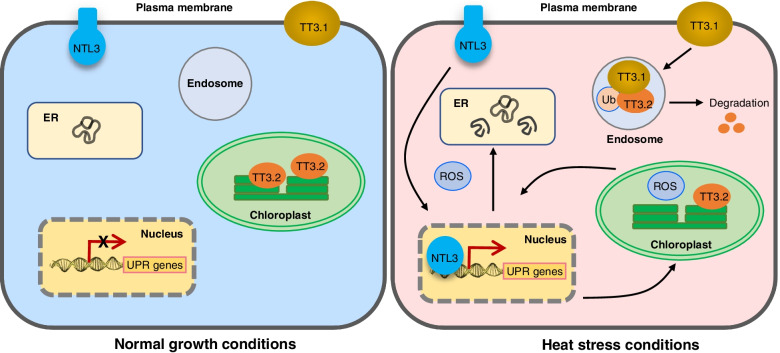
Translocating of thermosensors from plasma membrane (PM) to other organelles for thermotolerance in rice. Both TT3.1 and NTL3 are PM-localized proteins under normal growth temperature conditions. Upon heat stress, TT3.1 relocalizes from PM to endosomes to interact with and ubiquitylate TT3.2, leading to protein degradation of TT3.2 and relieve the damage effects of TT3.2 in chloroplasts. Meanwhile, NTL3 relocates to nucleus after proteolytic activation and regulates downstream genes to ensure proper protein folding in endoplasmic reticulum (ER). These PM-localized proteins might serve as thermosensors and confer thermotolerance in rice

In summary, Zhang et al. (2022) reported a novel QLT consists of *TT3.1* and *TT3.2* in rice. TT3.1 and TT3.2 forms a regulatory module to communicate among PM, endosomes and chloroplasts under heat stress conditions. In future studies, it would be exciting to find out how TT3.1 receives high temperature signals. *TT3.1* and *TT3.2* are conserved in other major crops such as maize and wheat, it would be interesting to know whether these orthologous genes could be used for breeding heat tolerant crops.

## Data Availability

Not applicable.
